# A deep neural network model for packing density predictions and its application in the study of 1.5 million organic molecules†
†Electronic supplementary information (ESI) available: It provides the SMILES of the virtual compound library, SAscores of the 15 building blocks, and details of the computational and experimental data underlying the figures and tables throughout this paper and that were used in the statistical analysis. We also provide the list of descriptors used to develop the DNN model and the trained model in scikit-learn's pickle (pkl) format. We note that this and other trained density models as well as the corresponding ML workflows are available as part of the *ChemML* package's template and model collection (*e.g.*, for retraining, customization, or transfer learning).^51^ Finally, we give detailed definitions of all statistical metrics employed in this work. See DOI: 10.1039/c9sc02677k


**DOI:** 10.1039/c9sc02677k

**Published:** 2019-07-09

**Authors:** Mohammad Atif Faiz Afzal, Aditya Sonpal, Mojtaba Haghighatlari, Andrew J. Schultz, Johannes Hachmann

**Affiliations:** a Department of Chemical and Biological Engineering , University at Buffalo , The State University of New York , Buffalo , NY 14260 , USA . Email: m27@buffalo.edu ; Email: hachmann@buffalo.edu; b Computational and Data-Enabled Science and Engineering Graduate Program , University at Buffalo , The State University of New York , Buffalo , NY 14260 , USA; c New York State Center of Excellence in Materials Informatics , Buffalo , NY 14203 , USA

## Abstract

The process of developing new compounds and materials is increasingly driven by computational modeling and simulation, which allow us to characterize candidates before pursuing them in the laboratory. One of the non-trivial properties of interest for organic materials is their packing in the bulk, which is highly dependent on their molecular structure. By controlling the latter, we can realize materials with a desired density (as well as other target properties). Molecular dynamics simulations are a popular and reasonably accurate way to compute the bulk density of molecules, however, since these calculations are computationally intensive, they are not a practically viable option for high-throughput screening studies that assess material candidates on a massive scale. In this work, we employ machine learning to develop a data-derived prediction model that is an alternative to physics-based simulations, and we utilize it for the hyperscreening of 1.5 million small organic molecules as well as to gain insights into the relationship between structural makeup and packing density. We also use this study to analyze the learning curve of the employed neural network approach and gain empirical data on the dependence of model performance and training data size, which will inform future investigations.

## Abstract 2

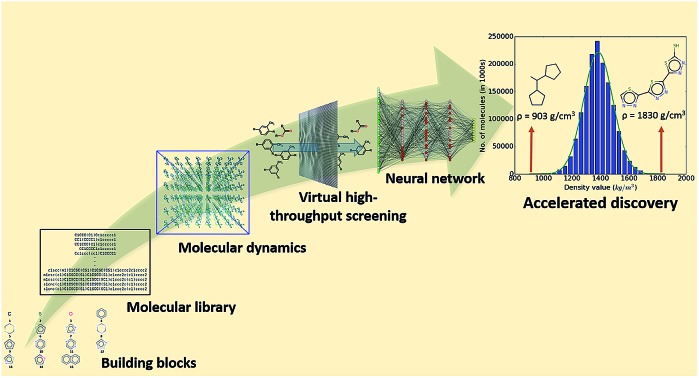
Computational pipeline for the accelerated discovery of organic materials with high refractive index *via* high-throughput screening and machine learning.

## I. Introduction

The packing of atoms, molecules, and polymers in a given volume – either in crystalline or amorphous form – is a fundamental and long-standing issue that has been considered by various disciplines for over a century.^1^ The packing density directly impacts properties such as the ionic conductivity,^2^ mobility in solvents,^3^ mechanical^4^ and optical behavior,^5^,^6^ and numerous other physical and chemical properties.^7^ Today, the packing density has gained renewed attention in the context of developing advanced materials that fulfill very specific property requirements. Molecular materials and polymers are of particular interest as their packing in the bulk is directly affected by their molecular structure.^8^ Manipulating and tailoring the latter offers many opportunities (and challenges) to achieve targeted density values.

Traditional, experimentally-driven trial-and-error searches for new compounds with desired sets of properties have proved to be time consuming and resource intensive. The advent of powerful modeling and simulation techniques as well as readily available time on high-performance computing systems have brought computational and computationally-guided studies to the forefront of the chemical and materials domain. These studies allow us to make increasingly accurate predictions for compounds of interest and uncover promising leads for experimentalist partners to follow up on (see, *e.g.*, ref. 9–20). An even more recent development has been the emergence of machine learning techniques that empower us to advance, augment, correct, or even replace physics-based modeling and simulation.^21^ In the latter scenario, machine learning is used to create data-derived property prediction models that serve as surrogates for physics-based models in order to dramatically accelerate the compound characterization and thus the overall discovery process. In addition to enabling hyperscreening studies, we can employ machine learning to gain a better understanding of the structure–property relationships that determine the behavior of compounds in the corresponding domains of chemical space. The creation of new machine learning prediction models for various target properties and the advancement of the underlying methodology is an active field of research.^22^ Key considerations are accuracy, cost, robustness, and range of applicability. Artificial neural networks are a popular and efficient machine learning approach.^23^ Multi-layer ‘deep’ neural networks (DNNs) yield particularly flexible models that have been used to predict an array of chemical properties, including refractive indices,^24^ dielectric constants,^25^ atomization energies,^26^ chemical reactivities,^27^ melting points,^28^,^29^ viscosities,^30^ solubilities,^31^ and others.

In this work, we develop a DNN prediction model for the packing density of small organic molecules in an amorphous bulk phase and conduct a hyperscreening of 1.5 million candidate compounds. Our interest in this target property originates from our ongoing *in silico* discovery and design efforts for polymers with high refractive index (RI)^32^–^34^ to be used in optic and optoelectronic applications.^35^,^36^ We previously established an RI modeling protocol based on the Lorentz–Lorenz equation and parametrized with the polarizability and number density.^32^,^33^ For the number density, we introduced a hybrid physics-based/data-derived prediction model using the van der Waals volume computed *via* the Slonimskii method and the packing coefficient from a support vector regression machine learning model.^37^ An alternative and commonly employed route to computing the (number) density, is the use of molecular dynamics (MD) simulations, which we recently started exploring in our study of high-RI polymers. However, as these MD calculations are computationally expensive and technically challenging, they are not particularly well suited for the large-scale assessment of compounds in the course of high-throughput screening studies. To bypass this problem, we develop a DNN surrogate model for the MD density predictions. It allows us to rapidly and accurately compute the density values of the 1.5 million molecules of a virtual screening library we create for proof of concept. For this, we perform MD simulations on a subset of 100 000 compounds, use the results to train our DNN model, and subsequently employ it to compute the packing density of the remaining 1.4 million molecules. We mine the density results to identify patterns that lead to desirable outcomes (*i.e.*, different density regimes). We also evaluate the learning curve for the density prediction to assess the dependence of training set size and model accuracy.

In Sec. II, we detail the methods employed in our work. We describe the MD modeling protocol we use to compute the density values (Sec. IIA), discuss the molecular design space we consider and the application of our virtual high-throughput screening tools on the resulting compound library (Sec. IIB), introduce our DNN prediction model (Sec. IIC), and establish our pattern analysis approaches to mine the obtained results (Sec. IID). Sec. III presents and discusses the outcomes of our study, in particular the density predictions from MD and DNN (Sec. IIIA), the efficiency of the DNN approach (Sec. IIIB), and the emerging structure–property relationships (Sec. IIIC). Our findings are summarized in Sec. IV.

## II. Methods and computational details

### A. Molecular dynamics modeling protocol

We employ the following MD modeling protocol to generate the data for the training and testing of the DNN density prediction model at the center of this work. Starting from the simplified molecular-input line-entry system (SMILES)^38^ string of a given compound, we employ the OpenBabel code^39^ to create a 3-dimensional structure guess, and then pre-optimize it using the MMFF94s force field^40^*via* steepest descent. We then compute the packing density with the general Amber force field (GAFF).^41^ For this, we obtain the GAFF parameters in automated fashion^42^ using the Antechamber toolkit that is part of AmberTools,^43^ and carry out the MD simulations within the GROMACS package.^44^ We employ GROMACS′ solvate tool to create a (10 nm)^3^ simulation box and fill it with the pre-optimized target molecules. The number of molecules in the simulation box depends on the given molecule size, but a typical system contains around 1000 molecules (*e.g.*, 972 for benzylcyclopentane). The system is first subjected to a minimization of the internal energy, which is associated with the relaxation of bonds, bond angles, and dihedral bond angles. This is followed by NVT and NPT equilibration steps for 100 and 240 ps, respectively. Both NVT and NPT ensembles use a Nosé–Hoover thermostat at 298.15 K for temperature control. The NPT ensemble uses the Parinello–Rahman barostat for pressure control. We conclude the MD protocol with a final 40 ps NPT production run. We use an MD timestep of 0.2 fs. We obtain the density by averaging the density values of the system at intervals of 0.2 ps during this final run. We note that this protocol is expected to yield kinetically stable amorphous phases rather than thermodynamically stable crystal structures or meta-stable polymorphs. GAFF is known to underestimate the density values compared to those from experiment, especially for high-density compounds.^45^ We employ a linear fit between the calculated and experimental values to account for the systematic differences and empirically calibrate the MD results.

### B. Candidate library generation and high-throughput screening

**Fig. 1:**
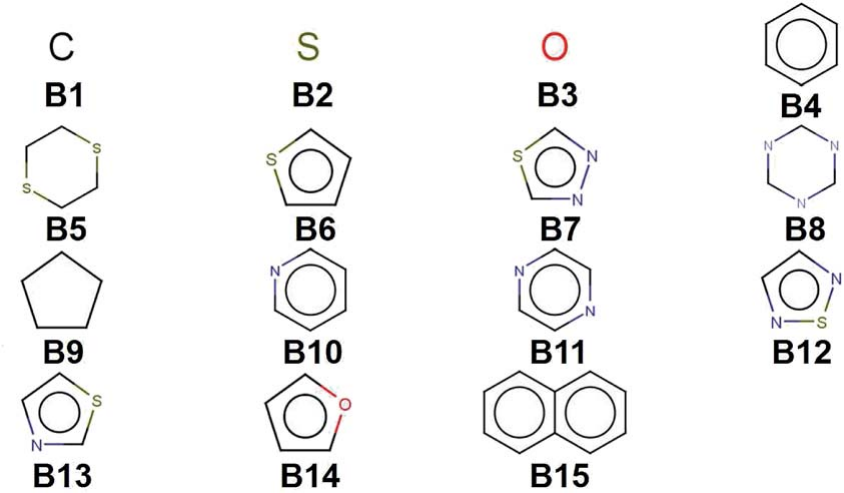
Molecular building blocks used to create the candidate library of 1.5 million compounds studied in this work.

We create a virtual library of 1.5 million small organic molecules using our library generator code *ChemLG*^46^,^47^ in constrained combinatorial mode. This library is constructed based on the sequential combinatorial linking of the 15 molecular building blocks shown in Fig. 1 for four generations, while enforcing certain constraints, *i.e.*, a molecular weight within the range of 150 to 400 Dalton and limiting the number of ring-moieties to four. The hydrogen atoms in each building block are used as linker handles. Our proof-of-principle library is designed to feature different connections between simple moieties, most of which are commonly used in organic materials (except **B5**, **B8**, and **B9**). We use the eToxPred software to compute the synthetic accessibility score (SAscore) of all 15 building blocks^48^ and obtain similarly favorable values between 2.4 and 2.9 on the 1–10 scale (with 1 being the most synthetically accessible). We provide the details of the accessibility analysis in the ESI.† Our generation approach limits the size of the library while yielding both a diverse set of compounds as well as candidates with more subtle differences for the model to distinguish. The complete library is provided in the ESI.†

To facilitate the density evaluation for a large number of compounds *via* the MD modeling protocol introduced in Sec. IIA, we employ our automated virtual high-throughput screening framework *ChemHTPS*.^46^,^49^ *ChemHTPS* creates inputs for the MD simulations, executes the modeling protocol, monitors the calculations, parses and assesses the results, and extracts and processes the information of interest. Of the 1.5 million compounds in our screening library, we randomly select a subset of 100 000 for study at the MD level.

### C. Neural network prediction model

We use the MD results for these 100,000 molecules as the ground truth for our data-derived density prediction model. For this, we pursue a DNN approach within a feature space of molecular descriptors. We build the DNN model using *ChemML*,^46^,^50^,^51^ our program suite for machine learning and informatics in chemical and materials research. In this work, *ChemML* employs the scikit-learn 0.18.2 library for the multi-layer perceptron regressor 1.17.1 (ref. 52) and 197 descriptors from Dragon 7.^53^ These descriptors include constitutional indices and functional group counts. If two descriptors are mutually correlated, they are not independent and thus redundant. In the cases where the Pearson correlation coefficient *R* is >95%, Dragon removes one of them, *i.e.*, the one that shows more correlation with the rest of the descriptors. (A detailed list of the descriptors is provided in the ESI.†) We apply the grid search method for a coarse optimization of the DNN model hyperparameters. The hyperparameter search space includes a number of activation functions (identity, tanh, rectified linear unit, and logistic), L2 regularization parameters (0.1, 0.01, 0.001, 0.0001, and 0.00001), solvers for the optimization of the weights (sgd and adam), and learning rate types (constant, invscaling, and adaptive). The best model from the hyperparameter optimization features the rectified linear unit as the activation function, ‘adam’ solver, adaptive learning rate, and an L2 regularization parameter of 0.0001. The final DNN has two fully connected hidden layers with 100 neurons each. For the initial model evaluation, we randomly divide the 100 000-molecule data set into 80% training and 20% test set. To assess the learning curve, we evaluate the model performance for incrementally increasing training set size from 0.05% to 100% of the entire data set (*i.e.*, from 50 to 100 000 data points). We apply the bootstrapping method, *i.e.*, for each training set size, we obtain the training set by randomly sampling the entire data set. The remaining data points serve as test set. For every training set size, we repeat the process (with replacement) 50 times, *i.e.*, all 50 repetitions are independent of each other. We subsequently calculate statistics over the results of the 50 models that are based on these training sets for each training set size.

### D. Data mining and pattern recognition

In addition to identifying candidates with particular density values from our MD screening and DNN hyperscreening studies, we mine the compiled results to better understand the correlation between molecular structure and packing density. One pattern recognition approach we pursue is the hypergeometric distribution analysis, in which we determine the *Z*-scores (*Z*_i_) of each building block i used in the creation of the molecular library as
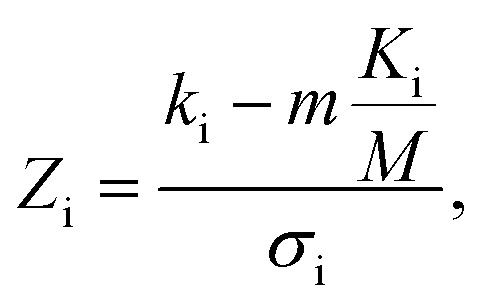
with
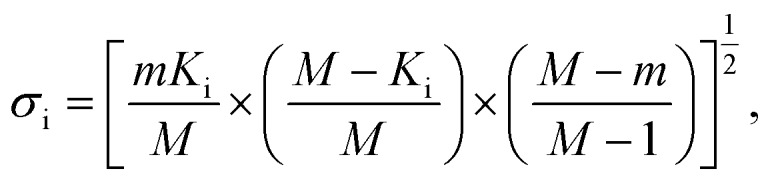
where *M* is the total number of molecules in the entire library, *m* is the subset of molecules under consideration (*e.g.*, the compounds in a certain density regime), *K*_i_ is the number of occurrences of building block i in *M* molecules and *k*_i_ its occurrences in the subset of *m* molecules. A large *Z*-score indicates that a building block appears more frequently in that subset compared to the rest of the library (or a random sample). By applying the hypergeometric distribution analysis, we can thus identify the building blocks with the largest impact on the target property and the degree to which they correlate with desired density values. Furthermore, we identify the building blocks that are prominent in particular density regimes and assess *Z*-score trends in density-ordered candidate subsets across the entire density range. In addition, we compute the average density values of the candidates derived from each building block, and analyze this data for trends. We employ the *ChemML* package for all data mining and pattern recognition tasks.

The following metrics are used in the error analyses of our modeling approaches: mean absolute error (MAE), mean absolute percentage error (MAPE), root mean squared error (RMSE), root mean squared percentage error (RMSPE), mean error (ME), mean percentage error (MPE), maximum absolute error (MaxAE), and maximum absolute percentage error (MaxAPE). Aside from providing these direct measures, we also quantify the extent of correlations and systematic biases between results of different methods by listing the correlation coefficients *R*^2^, slopes, and offsets of linear regressions.

## III. Results and discussion

### A. Density predictions

To test the accuracy of the MD density modeling protocol introduced in Sec. IIA, we compare its results against the experimentally known density values of 175 small organic molecules.^54^ This collection of compounds exhibits densities between 600 and 2000 kg m^–3^. As shown in the first column of Table 1, the calibrated MD results obtained for this collection are in good agreement with the experimental data, in particular considering that the density is also affected by factors other than the molecular structure (*e.g.*, processing and ambient conditions) that are not accounted for in the simulations. We also note that the experimental data set is structurally more diverse than the screening library, *i.e.*, it includes non-aromatic and aromatic moieties, halogens, and different functional groups such as OH, C=C, C≡C, C=O, N=O, *etc*. Despite this diversity, the comparison shows an *R*^2^ of 0.95, which underscores the utility of the employed MD approach. (Note that the very small ME/MPE as well as the ideal offset and slope are due to the empirical calibration scheme introduced in Sec. IIA. The calculated slope and offset of the linear fit between MD and experimental data is 0.84 and 121 kg m^–3^, respectively.)

**Table 1:** Performance comparison of our density predictions approaches. The column labeled MD (exp) compares the calibrated MD predictions with the experimental values of our collection of 175 compounds. The column labeled DNN (MD) compares the DNN predictions with the 20 000 MD results of our test set: all errors (MAE, RMSE, ME, MaxAE) and the offset are given in kg m^–3^

	MD (exp)	DNN (MD)
*R* ^2^	0.95	0.98
Slope	1.00	0.97
Offset	0.00	38.49
MAE (MAPE)	50.8 (4.7%)	10.8 (0.9%)
RMSE (RMSPE)	69.5 (6.2%)	13.6 (1.1%)
ME (MPE)	0.0 (0.0%)	–3.5 (–0.3%)
MaxAE (MaxAPE)	225.0 (20.4%)	59.2 (5.5%)

**Fig. 2:**
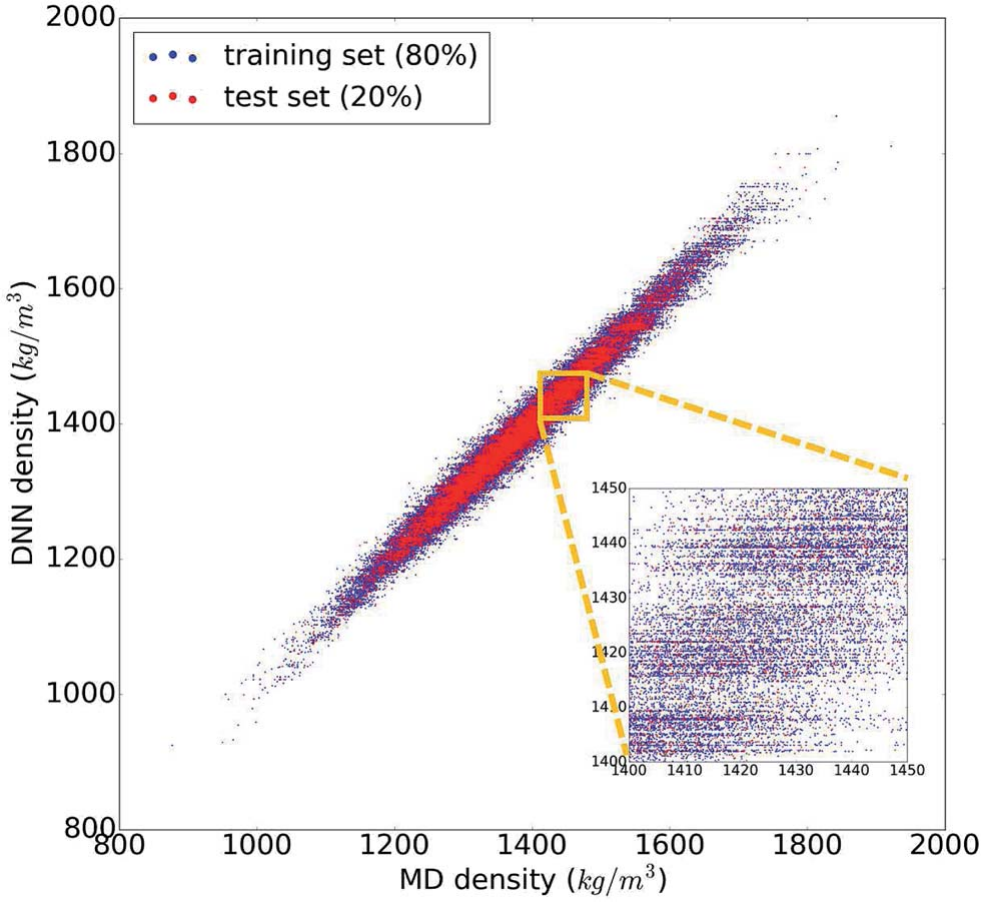
Comparison of the 100 000 calculated density values from molecular dynamics (MD) and the deep neural network (DNN) prediction model. Data points of the DNN training set are shown in blue and those of the test set in red.

The trained DNN model mimics – by design – the MD simulations it is derived from. The second column of Table 1 shows the DNN predictions for the MD test data. The benchmark analysis reveals a very good agreement between the MD and DNN results. The correlation coefficient is *R*^2^ = 0.98, both MAPE and RMSPE are around 1%, and MaxAPE is just 5.5%. Most importantly, we find that the DNN prediction errors are significantly smaller than the intrinsic MD errors (by a factor of 4 to 6), which means that the DNN and MD results are statistically indistinguishable. The prediction quality for the MD training data set is essentially identical to the test set shown in Table 1, indicating that our DNN model is not overfitted and that its predictions are sound and reliable. Fig. 2 shows the comparison of the MD and DNN results for the 100 000 compounds for which both MD and DNN results were computed with training data shown in blue and test data in red. We note that the inset of Fig. 2 shows clusters of molecules for which the DNN model predicts very similar density values (suggested by the horizontal lines of data points). This indicates a certain incompleteness of the selected feature representation and associated information loss, which suggests that even more accurate models can be achieved using other, more comprehensive descriptor sets (such as 3-dimensional molecular descriptors,^53^ extended connectivity,^55^ hashed topological torsion (HTT),^56^ hashed atom pair (HAP),^57^ or Morgan^58^ fingerprints, or a combination thereof). We also test our DNN model on the experimental data set. As the latter exhibits a structural diversity that goes well beyond the relatively narrow scope of the training data used to create the former, it yields unsurprisingly large errors (MAPE = 9.7%, RMSPE = 13.5%). Nonetheless, the *R*^2^ = 0.89 shows that the DNN model still captures the structure–property relationships to a certain degree, and given appropriate training data, DNN should deliver predictive models for those compound pools as well.

**Fig. 3:**
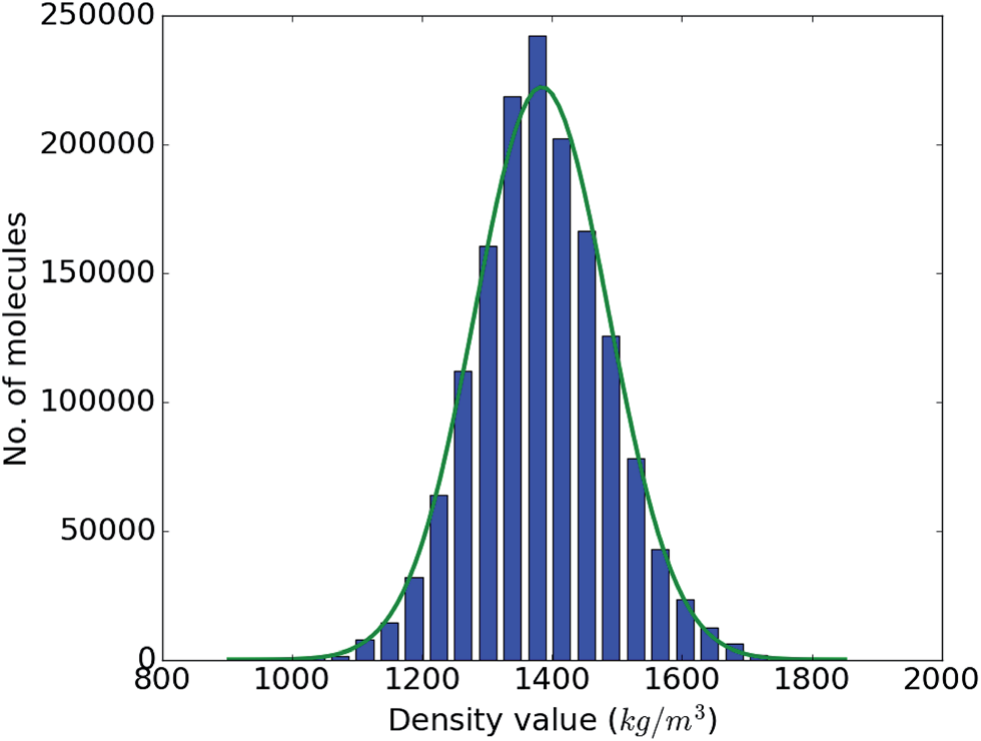
Range and distribution of the DNN density predictions for our proof-of-concept screening library of 1.5 million small organic molecules with a corresponding normal distribution overlayed.

With the accuracy of the trained DNN model established, we apply it to the remaining 1.4 million compounds of the screening library introduced in Sec. IIB with the expectation of obtaining similar results as MD would yield. The DNN density predictions are summarized in Fig. 3. The density values of the molecules at hand range from 902 to 1851 kg m^–3^ with an average of 1384 kg m^–3^. The results show a *t*- or Gaussian-like distribution and most of the compounds in the library have density values between 1200 to 1600 kg m^–3^, with only very few examples at the extreme high and low density regime. It is worth noting that these extreme packing density values may be desirable for certain material applications (*e.g.*, light-weight plastics with large strength-to-density ratio or rigid, impact-resistant thermo-plastics). The sparsity of instances for extreme density values emphasizes the valuable role that high-throughput screening studies *via* physics-based modeling and/or data-derived prediction models can play in the discovery of suitable materials candidates.

### B. Neural network efficiency

After confirming that the DNN prediction model can accurately reproduce MD-level results (which we in turn showed to accurately reproduce experimental data), we now investigate its efficiency, in particular relative to MD. Our MD calculations for the subset of 100 000 molecules took a total of 5 million core hours of compute time on a modern high-performance computing cluster. For the entire screening library, this extrapolates to approximately 75 million core hours (In addition to the compute time, there is generally a considerable amount of human time required for the setup and execution of these calculations. In our study, many of these tasks were performed by *ChemHTPS* without manual intervention.) The demand on disk space is another issue, and we estimate a need for 120 terabytes for the entire library (15 terabytes without trajectories). The DNN prediction model produces essentially the same results in less than 10 core hours of compute time (without performance optimization), with all but 10 minutes of the time required to generate the feature matrix of the compound library. Disk use is marginal. This corresponds to a speed-up of about seven orders of magnitude, with negligible loss in accuracy. A speed-up of that magnitude allows a corresponding increase in the scale and scope that is affordable for screening studies.

**Fig. 4:**
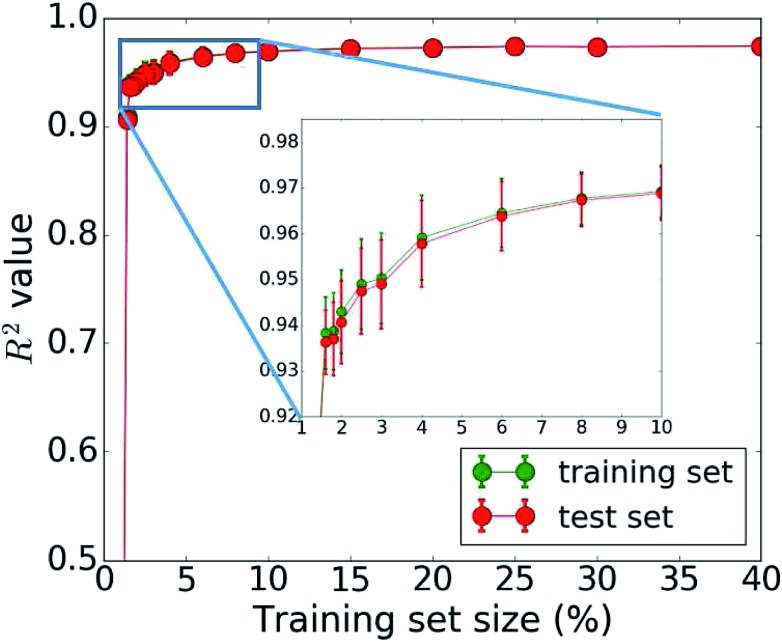
Dependence of the model accuracy (measured by the correlation coefficient *R*^2^) on the training set size (1% corresponds to 1000 data points). The learning curve shows the mean *R*^2^ from 50 bootstrap repetitions and the standard deviation is given in the error bars. The accuracy for the training set is plotted in green, that for the test set in red.

The bottleneck of our DNN prediction model is the generation of the training data needed for its creation. It is worth noting, though, that this is a fixed cost rather than an effort that scales with the number of compounds studied. The size of the employed training set (100 000 compounds corresponding to 5 million core hours) was originally chosen *ad hoc*. We now assess the learning curve as a function of training set size to gain insights into the actual data needs of our DNN model, which is one of the key questions in applying machine learning to any given problem setting. Our goal is to establish, how many data points are necessary to converge the learning process and/or achieve a desired accuracy. By minimizing the training set size requirement, we minimize the investment in computational resources needed to perform the expensive MD simulations. To address this question, we successively increase the size of the training set from 50 to 100 000 molecules. The resulting learning curve is shown in Fig. 4. We observe that all models trained on fewer than 2000 data points (*i.e.*, 2% of the available data) perform poorly. Models based on 2000 to 4000 data points offer acceptable accuracy. Those based on 4000 to 6000 data points offer very good accuracy, and at 10 000 data points, the training is essentially saturated and the learning curve plateaus off. Additional training data does not lead to an improvement of the DNN model and is essentially wasted. Thus, we do not require a large data set of 100 000 molecules to learn the packing density of organic molecules. We can develop an accurate model using MD data of just 5000 molecules (or more conservatively 10 000). This reduces our demand of computing time from 5 million to less than 0.25 (or 0.5) million core hours (including additional data for the test set), which has significant implications for the cost-benefit analysis and viability of this approach.

We stress that the data demand is highly dependent on the nature of the data and the employed machine learning approach (including the feature representation), and there are distinct limits to generalizing our findings. Instead of a *postmortem* analysis of the learning curve as provided here, we will use an on-the-fly assessment of the learning curve combined with a just-in-time termination of the training data generation to minimize our data footprint in future studies.

### C. Relationship between molecular structure and packing density

**Fig. 5:**
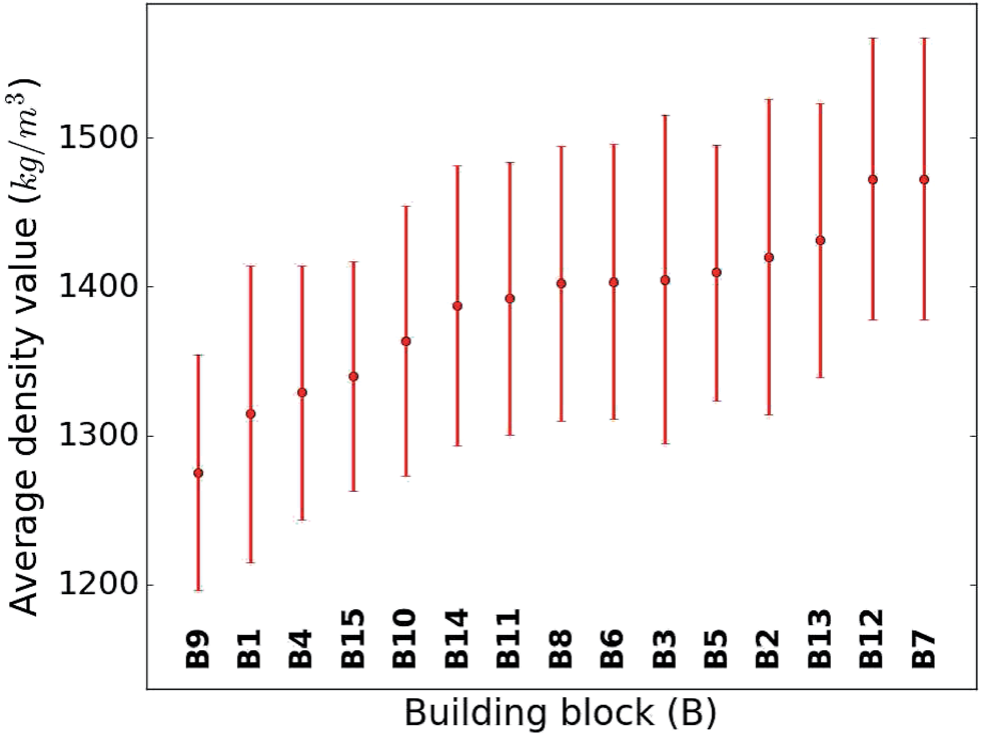
Density value distribution around the respective average density values (points) of the molecules containing a given building block. The bands refer to one standard deviation.

When considering the screening results, we are not only in a position to assess a large number of compounds, but we can also learn patterns from the data set in its entirety. Our analysis in Fig. 5 shows the average density values and distributions of all compounds containing a given building block (*cf.*Fig. 1). On the high density end, we find sulfur-heterocyclic moieties; the nitrogen- and oxygen-heterocycles yield medium density systems; and the low-density regime is dominated by carbon-based, non-heteroatomic building blocks. Molecules with **B7** (1,3,4-thiadiazole) and **B12** (1,2,5-thiadiazole) have the highest average densities, while those that incorporate **B1** (CH_2_-linker) and **B9** (cyclopentane) exhibit the lowest values. Aside from the linker groups, there is a clear correlation between density value and the heteroatom type and fraction in a corresponding moiety.

**Fig. 6:**
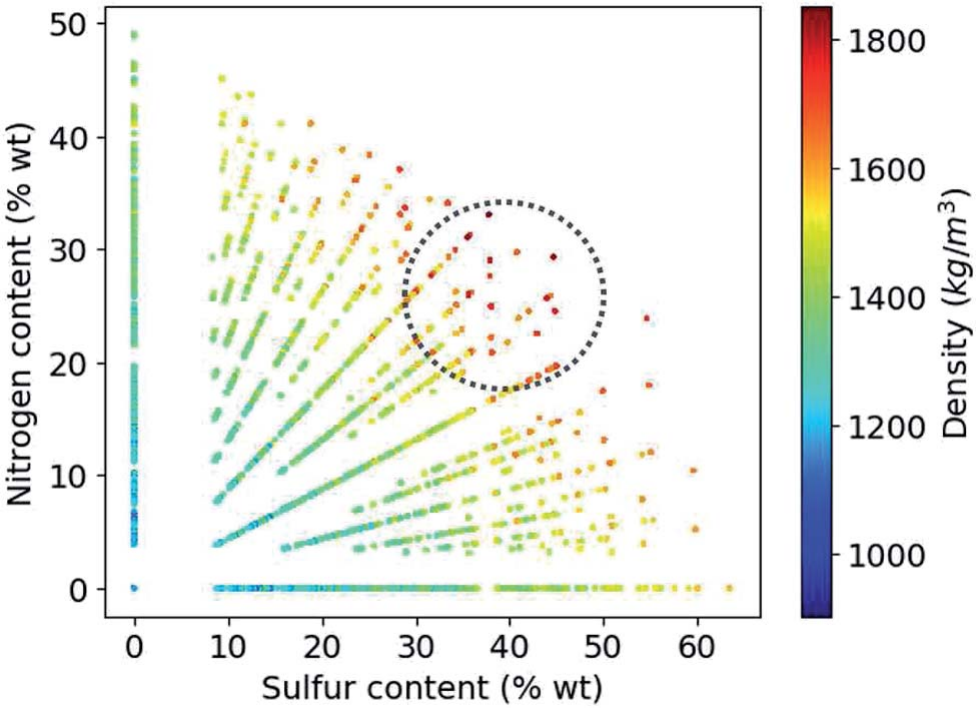
Variation of density values as a function of weight percentage of sulfur and nitrogen in the molecules.

Based on the construction of our library, more than 80% of the candidate compounds contain sulfur and more than 90% contain nitrogen. Fig. 6 demonstrates how the density values depend on the weight percentage of the sulfur and nitrogen atoms in the compounds at hand. Our library thus yields the highest density values for molecules that by weight contain 30 to 50% sulfur and 20 to 30% nitrogen.

**Fig. 7:**
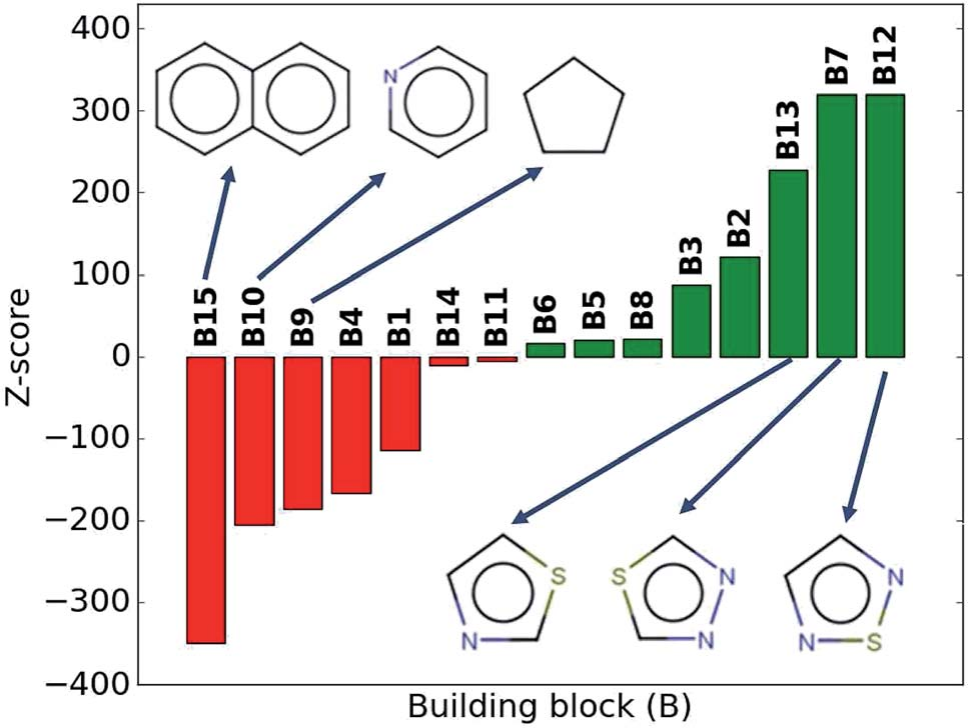
*Z*-scores of each building block in the compounds with the highest density values (top 10% of the library). Green represents positive *Z*-scores, and negative ones are shown in red.

**Fig. 8:**
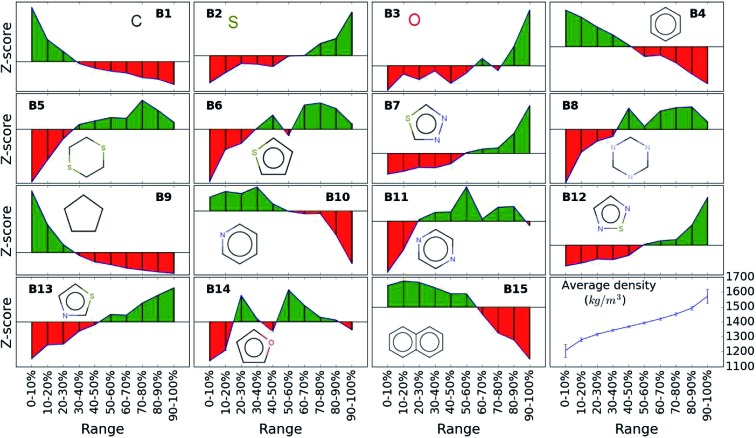
*Z*-score of each building block in all library subsets with increasing density values. Green color indicates positive *Z*-scores and red negative values. The last cell shows the average density values in each of the ten segment with increasing trend from 1200 to 1600 kg m^–3^.

While the average density values indicate the cumulative impact of a particular building block, we find relatively large standard deviations (*cf.*Fig. 5). For a more detailed picture of the occurrences of building blocks in a particular subset of the library, we perform the *Z*-score analysis introduced in Sec. IID. Fig. 7 shows the corresponding results for the molecules with the highest density values (*i.e.*, the top 10% subset) with clear and distinct trends. Consistent with our previous analysis, we observe very large *Z*-score values for and thus a strong overexpression of **B7** and **B12**. **B13** (thiazole) also shows a large *Z*-score, and so do to a lesser extent **B2** (S-linker) and **B3** (O-linker) as well. These moieties are clearly favorable if high-density compounds are desired. In addition to assessing the high-density regime, we employ the hypergeometric distribution analysis to identify the prevalence of building blocks in the complete spectrum of density values. For this, we sort our virtual library by increasing density values, divide it into ten equal segments, and perform our analysis within each of these subsets as shown in Fig. 8. Based on the data from this analysis, we can identify trends in the impact of individual building blocks on the density of organic molecules. The *Z*-score of building blocks **B2**, **B7**, **B12**, and **B13** increases with increasing density values, indicating a direct correlation, whereas it decreases for **B1**, **B4**, **B9**, **B10**, and **B15**, indicating an inverse correlation. The former are thus suitable to design organic molecules with higher density, and the latter could be used to achieve compounds with lower density. These findings are consistent with our prior analysis.

## IV. Conclusions

The ability to predict the properties of novel compounds prior to synthesis, and to understand how these properties depend on their structure, is of considerable importance in materials discovery and design. In this paper, we showed that MD simulations can accurately predict experimental packing density values of small organic molecules and we provided corresponding benchmark results to quantify this finding. We conducted a high-throughput MD screening of 100 000 compounds, which allowed us to train a DNN density prediction model. This DNN model accurately reproduces the MD data within the margins of MD's intrinsic error, while being nearly seven orders of magnitude faster than MD. This exceedingly efficient approach allowed us to rapidly obtain the density values of a 1.5 million compound screening library, which would have been prohibitively time consuming and well out of reach for MD. By analysing the large data set resulting from this study, we could elucidate structure–property relationships that determine the density values. We identified prevalent moieties in the high and low density regime and could quantify the impact of heteroatoms (sulfur and nitrogen). Further, we evaluated the DNN learning curve for the density prediction with respect to the available training data and found a considerably lower data demand than we had anticipated. Following this lesson, we will in future studies employ an on-the-fly assessment of the learning curve and terminate the training data generation once we observe satisfactory saturation. This will allow us to alleviate the data generation bottleneck and make machine learning models an even more viable and attractive proposition. Overall, our study underscores the value of combining powerful machine learning approaches with traditional computational modeling for the generation of the necessary data. It also demonstrates the utility of our software ecosystem (including the *ChemLG* molecular library generator code, the *ChemHTPS* automated high-throughput *in silico* screening program, and the *ChemML* machine learning package) in facilitating and supporting research efforts of this nature.

## Conflicts of interest

The authors declare to have no competing financial interests.

## Supplementary Material

Supplementary information

Supplementary information

Supplementary information
